# Supplementary material for: The influence of HLA genetic variation on plasma protein expression
Source: Nat Commun. 2024 Jul 31;15:6469. doi: 10.1038/s41467-024-50583-8 (PMC11291675; doi:10.1038/s41467-024-50583-8)
Supplement: Supplementary file 1 — Supplementary Information [file 41467_2024_50583_MOESM1_ESM.pdf]

Supplementary Information for

The influence of HLA genetic variation on plasma protein expression

Chirag Krishna[1], Joshua Chiou[1], Saori Sakaue[2-5], Joyce B. Kang[2-5], Stephen M. Christensen[1], Isac Lee[1], Melis Atalar Aksit[1], Hye In Kim[1], David von Schack[1], Soumya Raychaudhuri[2-5], Daniel Ziemek[1], Xinli Hu[1]*

*Corresponding author. xinli.hu@pfizer.com

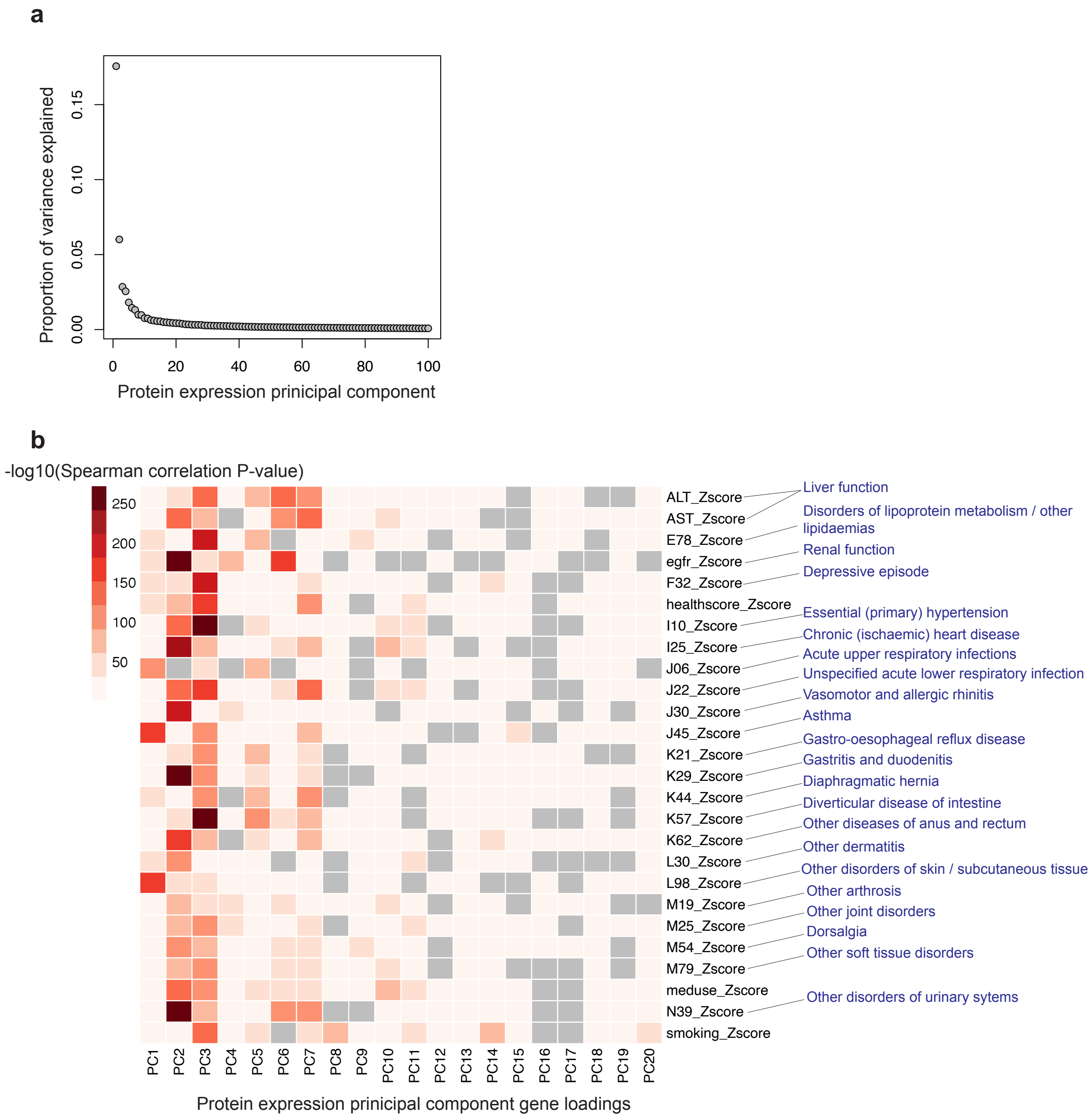

Supplementary Fig. 1: Principal component analysis of plasma protein expression in the UK Biobank.

a, Proportion variance explained by the top 100 principal components (PCs) of protein expression in the discovery cohort. The top 20 protein expression PCs were used as covariates in the HLA-pQTL linear models. **b**, Spearman correlation of the gene loadings for top 20 protein expression PCs with gene – disease associations (represented as Z-scores) computed by the UKB-PPP, for the most prevalent diseases in the discovery cohort. Gray indicates a non-significant (P-value > 0.05) association. Heatmap rows depict ICD10 code; blue text indicates description of ICD-10 code. Analyses were conducted in the discovery cohort with N = 34490 individuals.

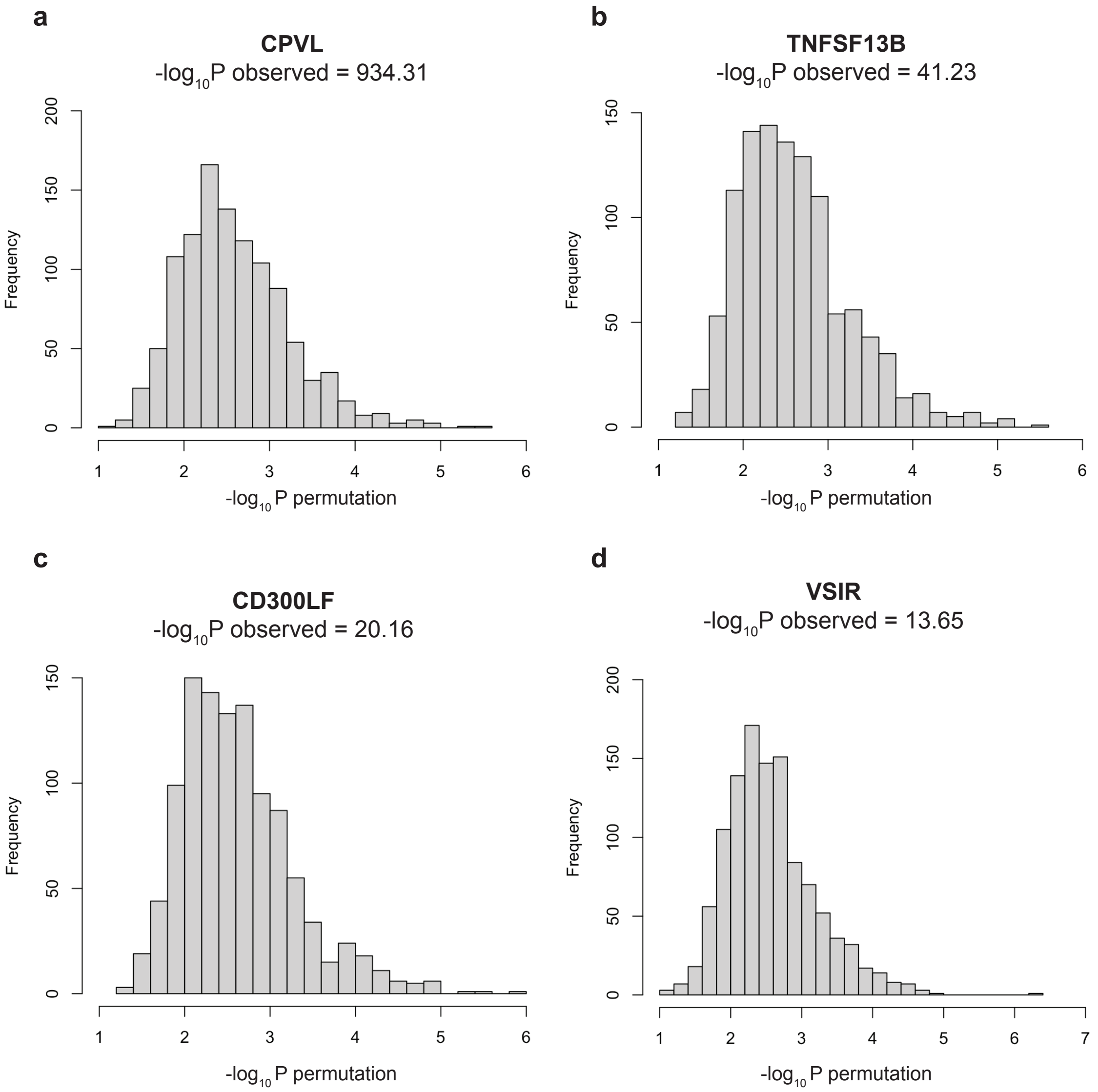

Supplementary Fig. 2: Permutation analysis to assess robustness of lead HLA-pQTL in the discovery cohort.

a-d, For 4 selected proteins, histograms of -$\log_{10}$ permutation p-values in which protein expression values were randomized across participants in the discovery cohort. For each permutation iteration (1000 total for each protein), all HLA variants were tested, and the lead variant (variant with minimal p-value) was retained to construct the null distribution plotted here. For all proteins, no permutation iterations achieved a -$\log_{10}$ permutation p-value greater than the observed -$\log_{10}$ p-value, listed for each protein. Analyses were conducted in the discovery cohort with N = 34490 individuals.

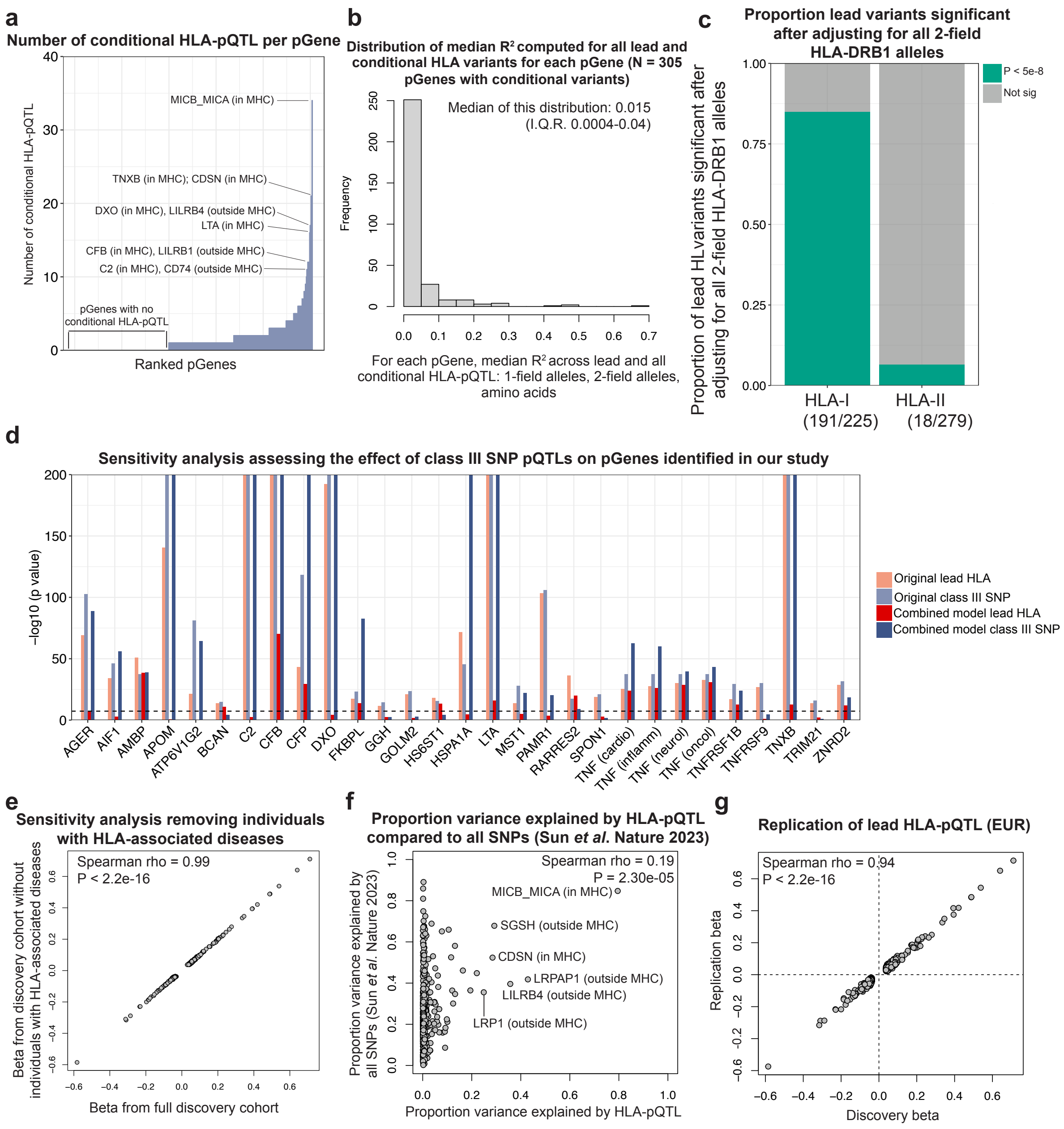

Supplementary Fig. 3: HLA-pQTL characteristics and sensitivity analyses in the discovery cohort.
a, Number of conditional HLA-pQTL across all HLA-pGenes. **b**, Distribution of median LD R^2 for each HLA-pGene between its lead HLA variant and all conditional HLA variants. **c**, Proportion of lead HLA-I and lead HLA-II pQTL that remained significant after adjusting for all 2-field HLA- DRB1 alleles. Parentheses beneath bars indicate fraction of significant lead pQTL remaining, equivalent to the proportions show in the bars. **d**, HLA-pGenes with significant SNP pQTLs in the HLA class III region from the flagship study. Barplot depicts significance of HLA-pQTL from our study, class III pQTL from the flagship study, and the significance of both in a combined model combining both our HLA-pQTL and the class III SNP. **e**, Concordance of effect sizes between lead HLA-pQTL in the full discovery cohort and lead HLA-pQTL in a reduced discovery cohort removing individuals with

HLA-associated diseases. **f**, Proportion of protein expression variance explained by lead and conditional HLA-pQTL compared to proportion of protein expression variance explained by all SNPs from the flagship UKB-PPP analysis. **g**, Concordance of effect sizes for HLA-pQTL that were nominally significant ($P < 0.05$) in the EUR replication cohort.

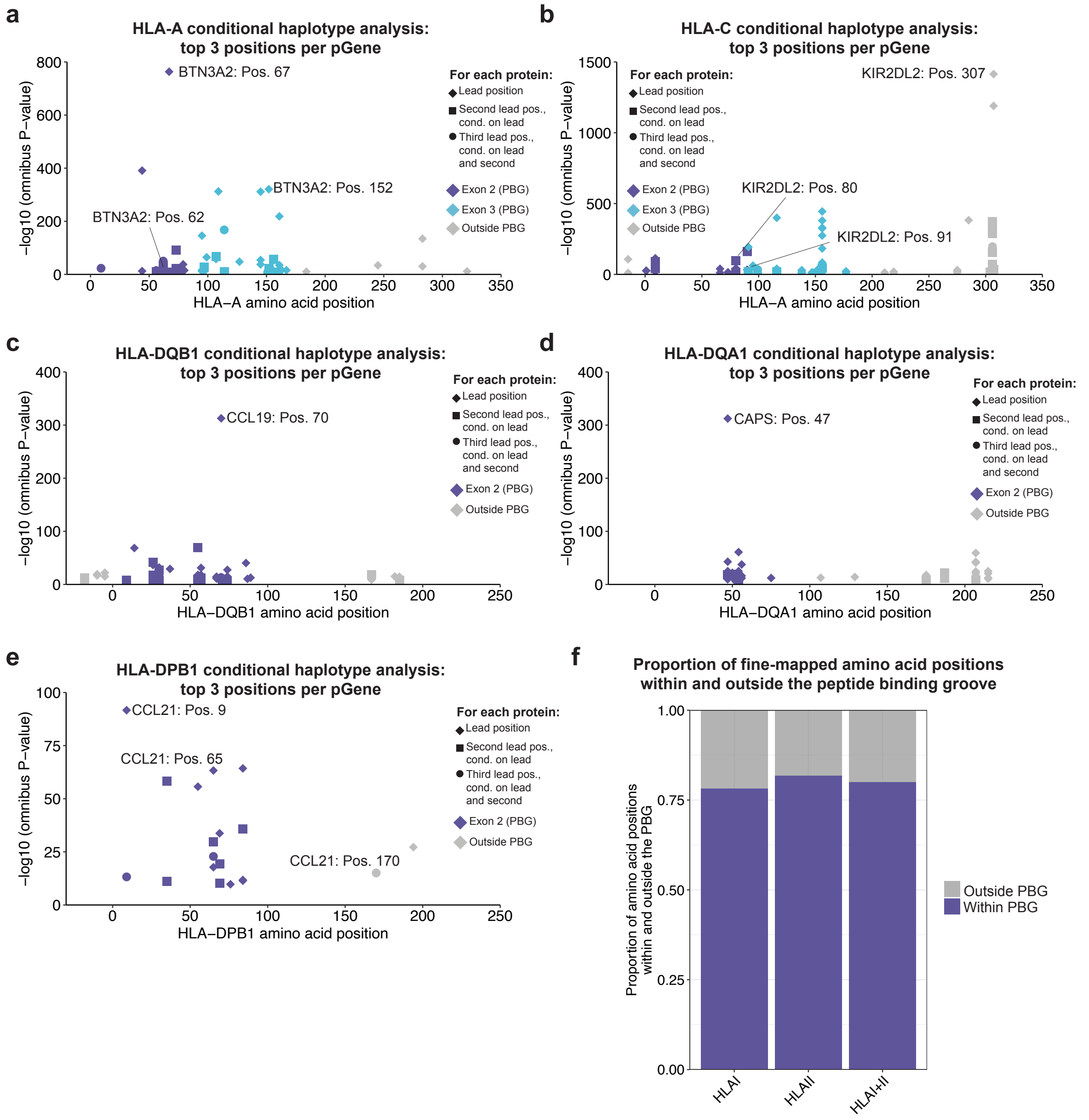

Supplementary Fig. 4: Significant HLA-pQTL identified via conditional haplotype analysis for loci not shown in Fig. 1.

a-e, Top three significant ($P < 5.0 \times 10^{-8}$) amino acid positions identified via conditional haplotype analysis shown for each locus, where available. The protein with lowest P-value across all proteins tested is labeled together with its top 3 significant conditional positions. **f**, Proportion of amino acids from conditional haplotype analysis within and outside the peptide binding groove. Analyses were conducted in the discovery cohort with N = 34490 individuals.

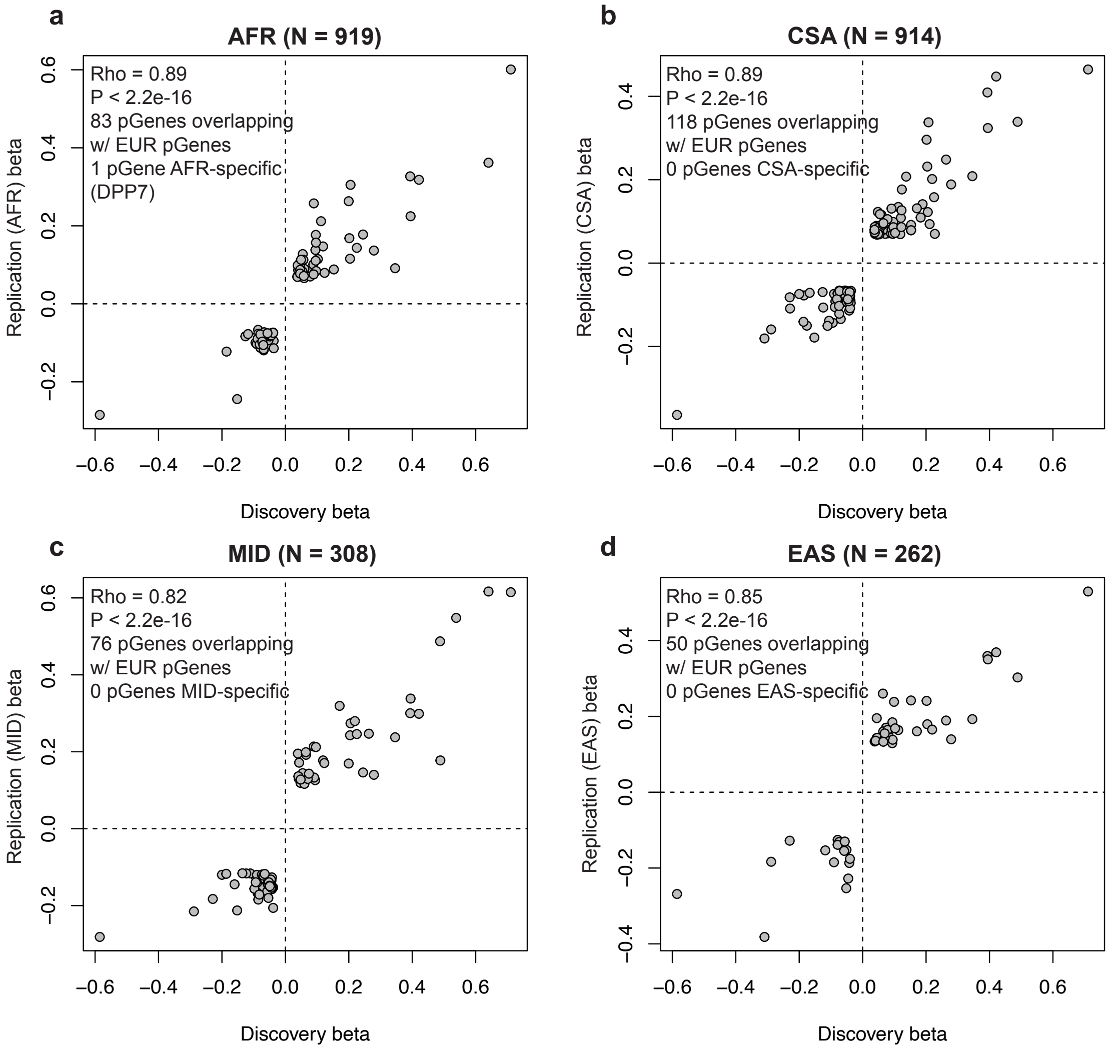

Supplementary Fig. 5: Multi-ancestry HLA-pQTL replication analyses.
a-d, Concordance of effect sizes for HLA-pQTL that were nominally significant (P < 0.05) in additional genetic ancestry-specific subgroups

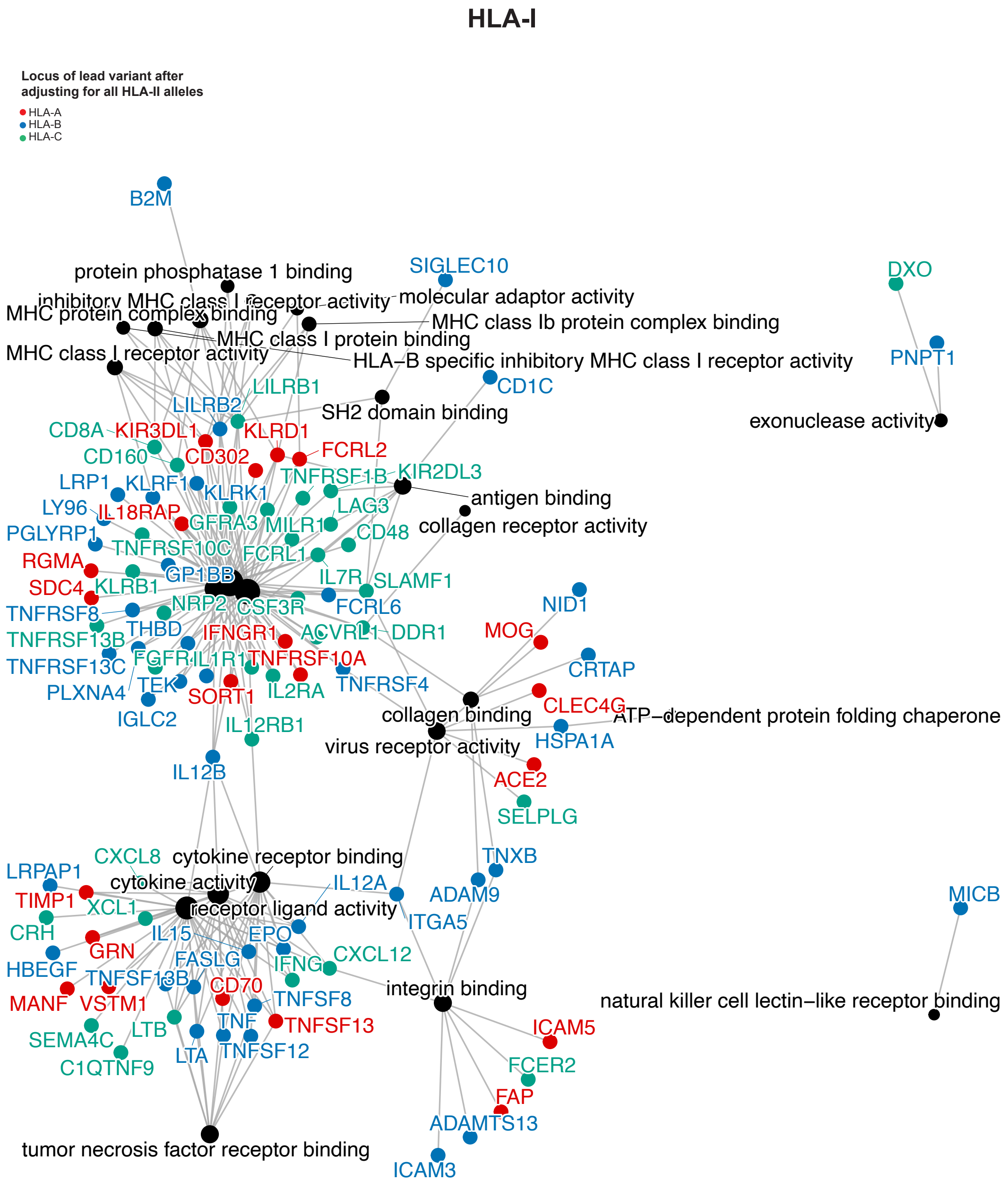

Supplementary Fig. 6: **Additional trans HLA-pQTL networks and gene families affected by HLA-I genetic variation.**
Data show all pathways significant at FDR P < 0.1. Analyses were conducted in the discovery cohort with N = 34490 individuals.

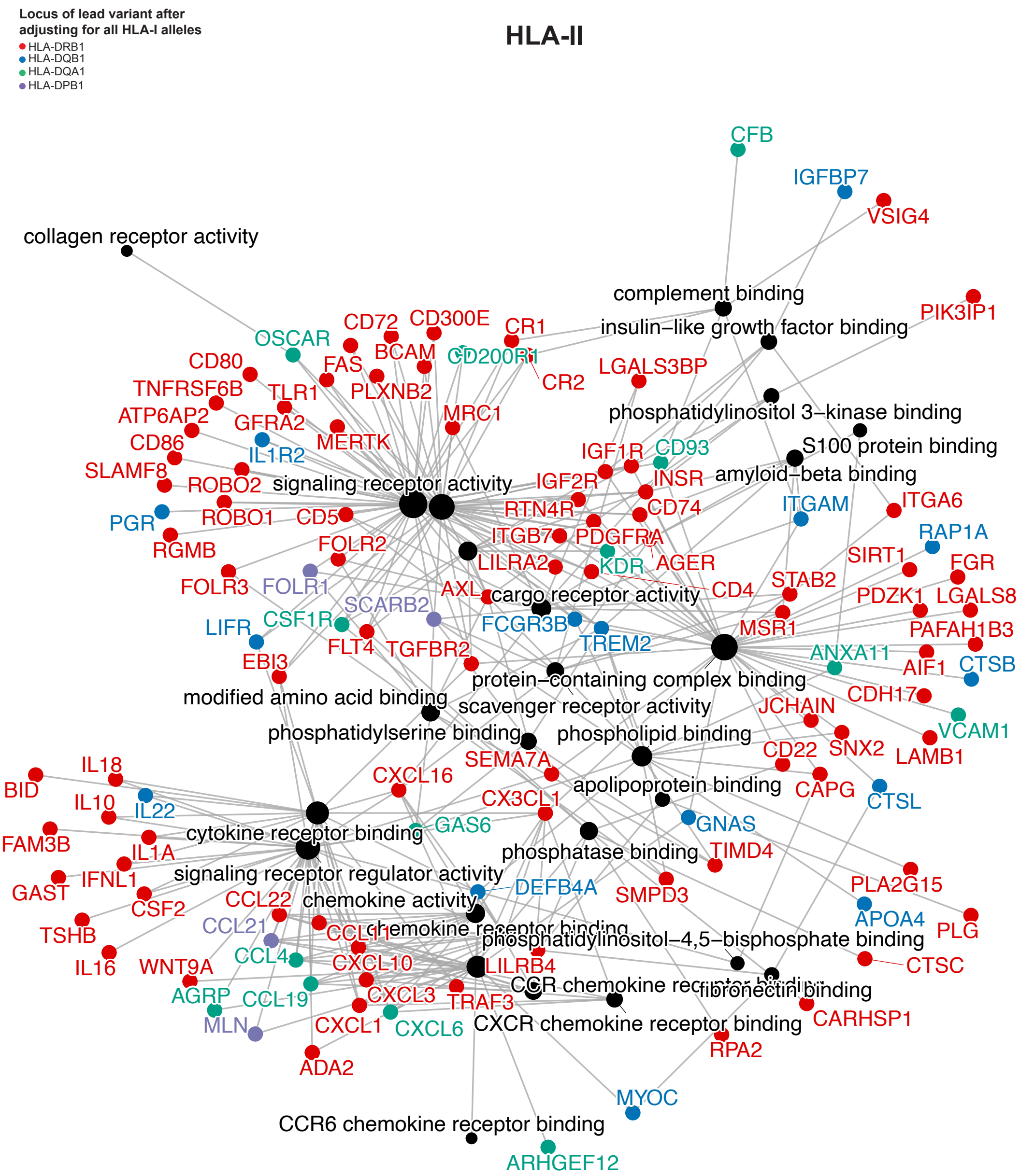

Supplementary Fig. 7: **Additional trans HLA-pQTL networks and gene families affected by HLA-II genetic variation.**

Data show all pathways significant at FDR P < 0.1. Analyses were conducted in the discovery cohort with N = 34490 individuals

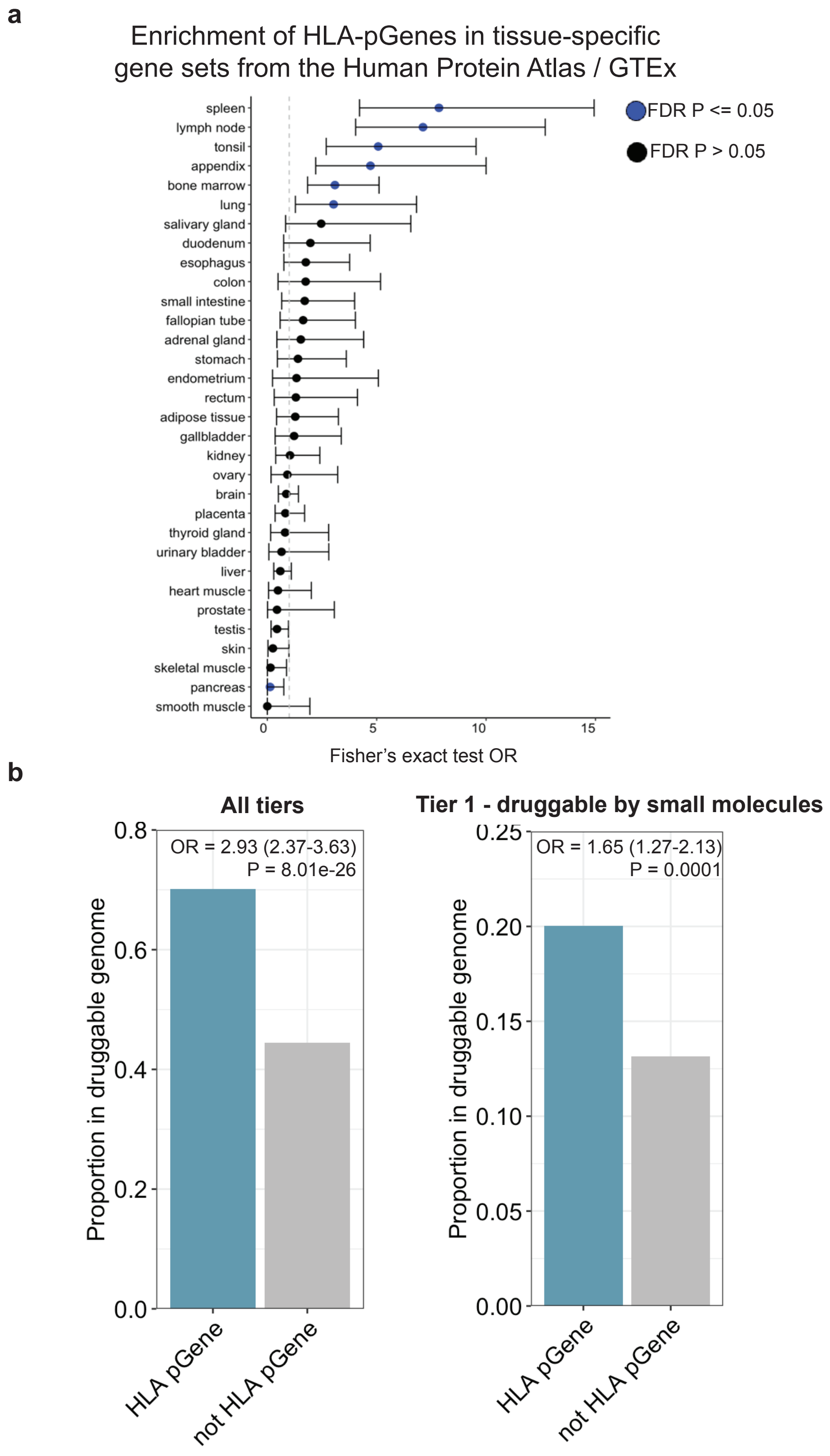

Supplementary Fig. 8: Enrichment of HLA-pGenes in tissue-specific gene sets (a) or genes from the druggable genome (b).

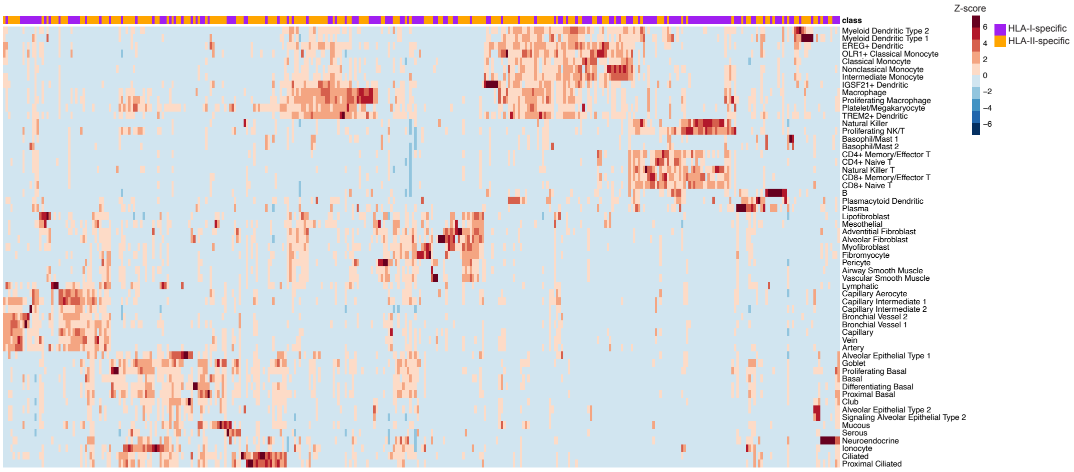

Supplementary Fig. 9: Expression of HLA-pGenes on all clusters (immune + non-immune) from the Travaglini *et al*. lung atlas.

a

cis-pQTL from flagship study	HLA-pGene from our study	GWAS catalog study ID	Disease
rs4939490	CD5	GCST000949	Multiple Sclerosis
rs4939490	CD5	GCST009597	Multiple Sclerosis
rs11230563	CD6	GCST005537	Chronic inflammatory diseases
rs11230563	CD6	GCST004131	Inflammatory bowel disease
rs11230563	CD6	GCST003043	Inflammatory bowel disease
rs11230563	CD6	GCST001725	Inflammatory bowel disease
rs11230563	CD6	GCST003044	Crohn's disease
rs11230563	CD6	GCST003045	ulcerative colitis
rs4129267	IL6R	GCST005537	Chronic inflammatory diseases
rs4129267	IL6R	GCST005529	Ankylosing spondylitis
rs6897932	IL7R	GCST001198	Multiple sclerosis
rs6897932	IL7R	GCST90270940	Systemic lupus erythematosus (MTAG)
rs34687326	SLAMF8	GCST004132	Crohn's disease
rs2062305	TNFSF11	GCST000879	Crohn's disease

b

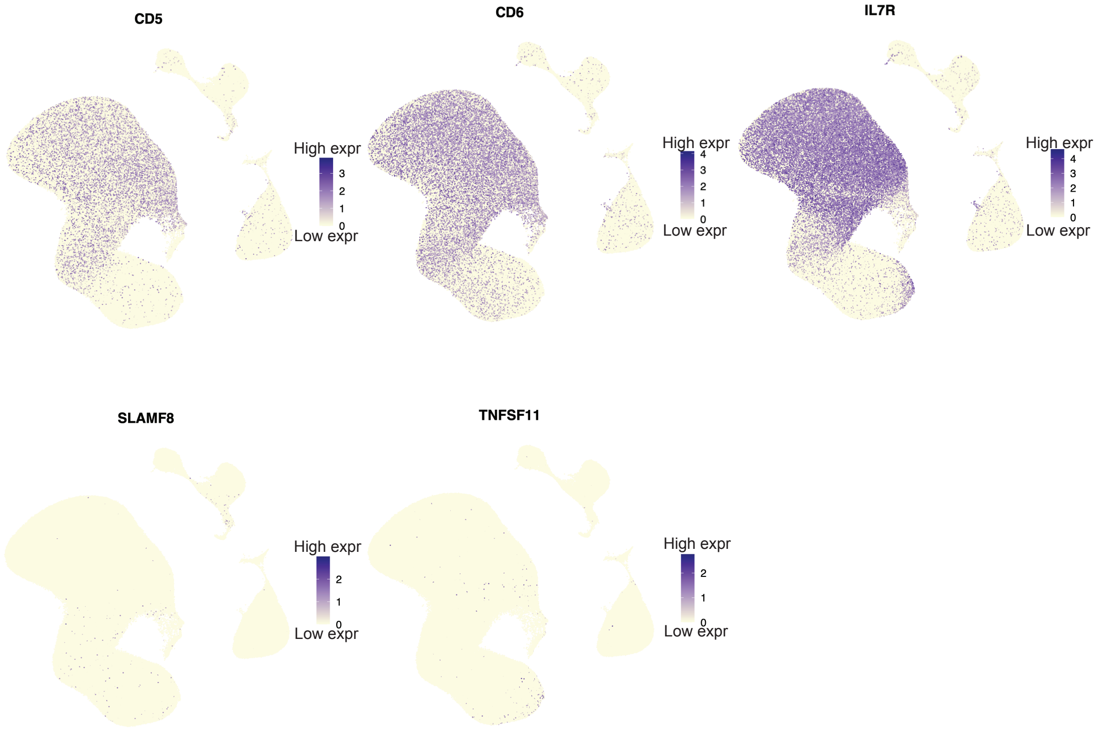

Supplementary Fig. 10: cis-pQTLs of HLA-pGenes associated (P < 5 x 10^{-8} from the GWAS catalog) with immune-mediated diseases (a) and expression of the corresponding HLA-pGenes on the Yazar *et al*. immune cell atlas.

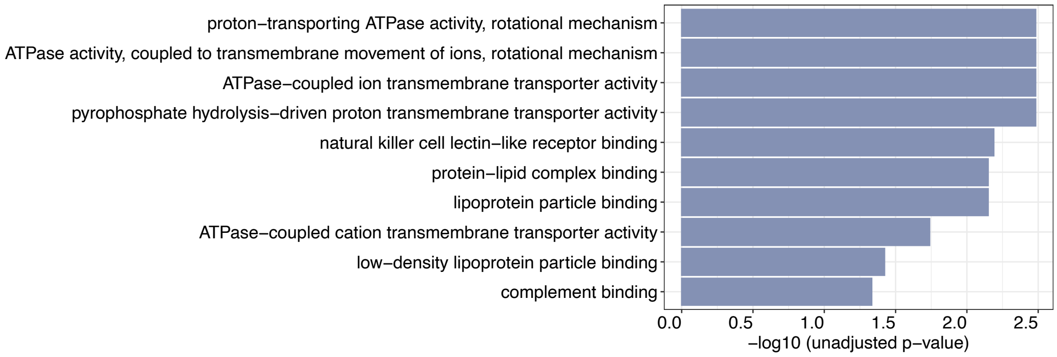

Supplementary Fig. 11: Gene ontology enrichment analysis depicting pathways enriched in trans, non HLA-pQTL at unadjusted P < 0.05. No pathways were significant at FDR P < 0.05.

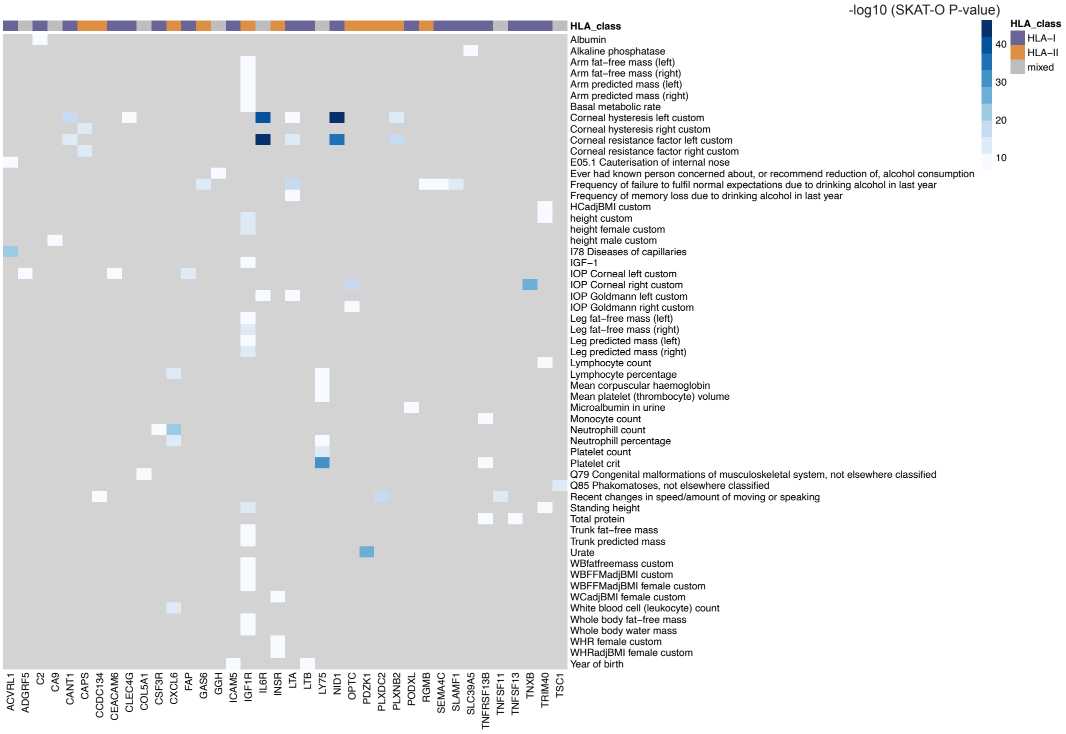

Supplementary Fig. 12: Disease associations for HLA-pGenes obtained via burden tests using genetic variation from exome sequencing.

Heatmap shows significant (SKAT-O P < 2.5 x 10^{-7}) associations from the genebass browser. Gray indicates non-significant association. Color bar along top of heatmap indicates whether pGene is HLA-I-specific, HLA-II-specific, or neither (‘mixed’).

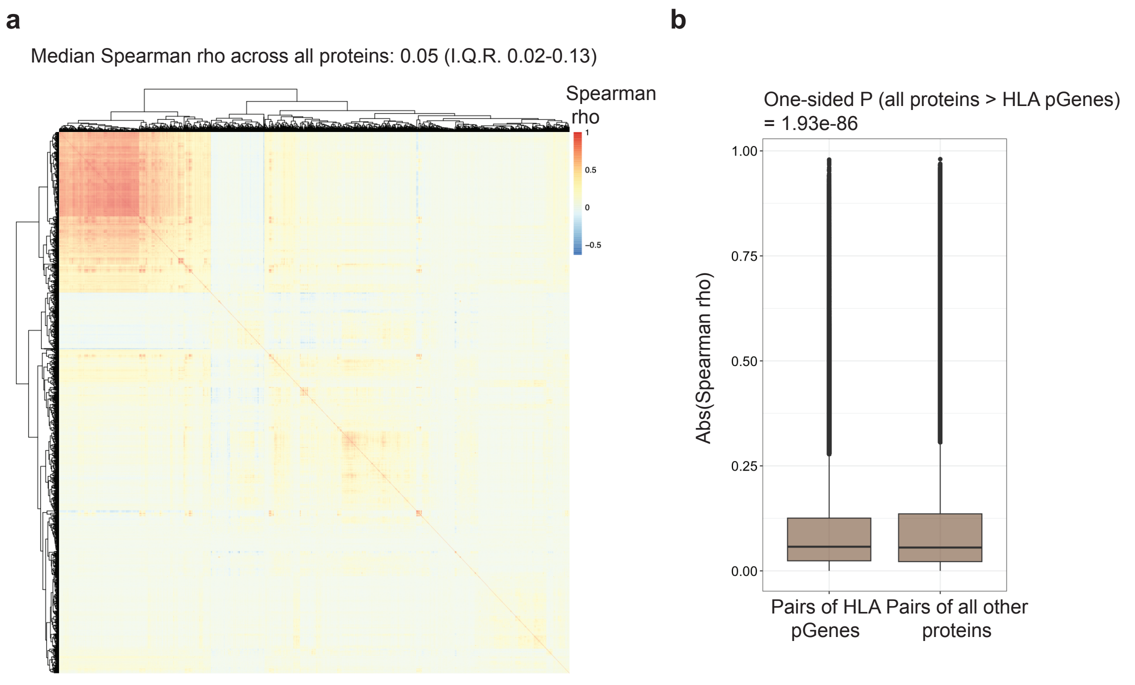

Supplementary Fig. 13: Pairwise protein correlations.
a, Pairwise Spearman correlations computed across all 2940 proteins. **b**, Spearman correlations for HLA-pGenes compared to all other pGenes. P-value calculated using one-sided Wilcoxon test.
