# Supplementary material for: The influence of HLA genetic variation on plasma protein expression
Source: Nat Commun. 2024 Jul 31;15:6469. doi: 10.1038/s41467-024-50583-8 (PMC11291675; doi:10.1038/s41467-024-50583-8)
Supplement: Supplementary file 3 — Description of Additional Supplementary Files [file 41467_2024_50583_MOESM3_ESM.pdf]

Description of Additional Supplementary Files

File Name: Supplementary Data 1
Description: Lead single-marker HLA-pQTL from the UKB-PPP discovery cohort

File Name: Supplementary Data 2
Description: Conditional single-marker HLA-pQTL from the discovery cohort

File Name: Supplementary Data 3
Description: Lead single-marker HLA-pQTL from the discovery cohort after conditioning on class III SNP pQTLs

File Name: Supplementary Data 4
Description: Lead single-marker HLA-pQTL from the discovery cohort after removing individuals with HLA-associated diseases, and list of diseases excluded

File Name: Supplementary Data 5
Description: Significant multiallelic amino acid positions from omnibus and conditional haplotype tests in the discovery cohort

File Name: Supplementary Data 6
Description: Nominally significant HLA-pQTL in the UKB-PPP EUR replication cohort

File Name: Supplementary Data 7
Description: Nominally significant HLA-pQTL in the UKB-PPP AFR replication cohort

File Name: Supplementary Data 8
Description: Nominally significant HLA-pQTL in the UKB-PPP CSA replication cohort

File Name: Supplementary Data 9
Description: Nominally significant HLA-pQTL in the UKB-PPP MID replication cohort

File Name: Supplementary Data 10
Description: Nominally significant HLA-pQTL in the UKB-PPP EAS replication cohort

File Name: Supplementary Data 11
Description: Lead HLA-pQTL from the combined UKB-PPP discovery and replication cohorts

File Name: Supplementary Data 12
Description: HLA-I-specific pGenes

File Name: Supplementary Data 13
Description: HLA-II-specific pGenes

File Name: Supplementary Data 14
Description: Gene ontology enrichment results for HLA-I-specific pGenes

File Name: Supplementary Data 15
Description: Gene ontology enrichment results for HLA-II-specific pGenes

File Name: Supplementary Data 16
Description: Normalized count matrix of HLA-I-specific and HLA-II-specific and clusters from the Yazar *et al*. immune cell atlas

File Name: Supplementary Data 17
Description: Normalized count matrix of HLA-I-specific and HLA-II-specific and clusters from the Travaglini *et al*. lung cell atlas

File Name: Supplementary Data 18
Description: Lead single-marker HLA-pQTL from the discovery cohort after conditioning on cis-eQTLs of HLA-pGenes

File Name: Supplementary Data 19
Description: Conditional HLA-pQTL from the discovery cohort based on conditional analysis of one- and two-field alleles only (to support **Table 1**)

File Name: Supplementary Data 20
Description: Abbreviations for all UKB traits linked to fine-mapped HLA-pQTL (to support **Fig. 4**)

File Name: Supplementary Data 21
Description: HLA-pGenes overlapping with genes from the druggable genome project
